# Correction: Tyramine induces dynamic RNP granule remodeling and translation activation in the *Drosophila* brain

**DOI:** 10.7554/eLife.70755

**Published:** 2021-05-28

**Authors:** Nadia Formicola, Marjorie Heim, Jeremy Dufourt, Anne-Sophie Lancelot, Akira Nakamura, Mounia Lagha, Florence Besse

Formicola N, Heim M, Dufourt J, Lancelot A-S, Nakamura A, Lagha M, Besse F. 2021. Tyramine induces dynamic RNP granule remodeling and translation activation in the *Drosophila* brain. *eLife* **10**:e65742. doi: 10.7554/eLife.65742.

Published 23, April 2021

In the published article, Figure 2C inadvertently presented four times the image corresponding to the first time point (t=0) of the control movie. This has been corrected and the proper image sequence is now shown. No further changes were made to the text and figure legend. Please note that this correction does not affect the results and conclusions of the original paper.

The corrected Figure 2C is shown here:

**Figure 1:**
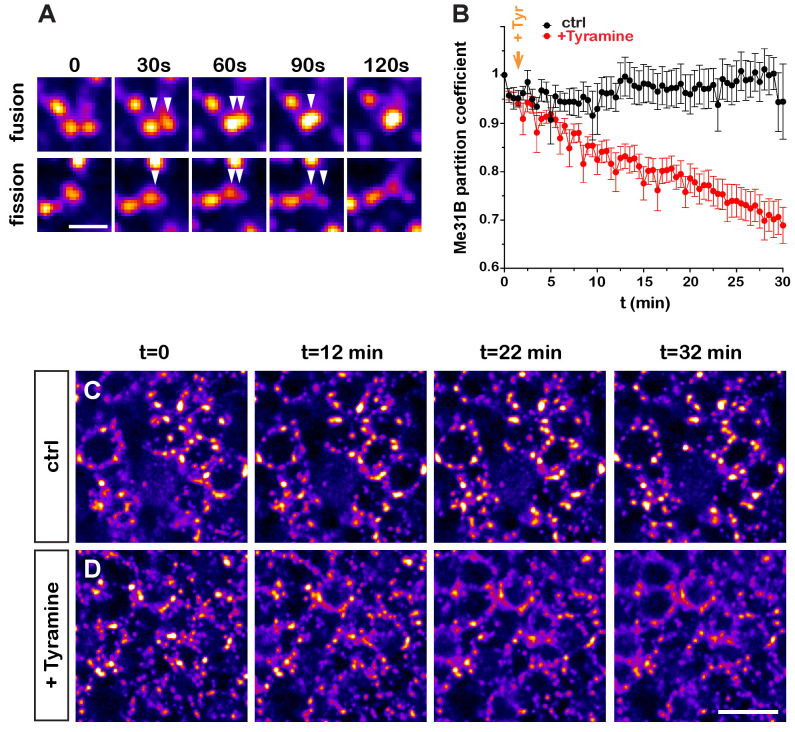


The originally published Figure 2C is also shown for reference:

**Figure 2:**
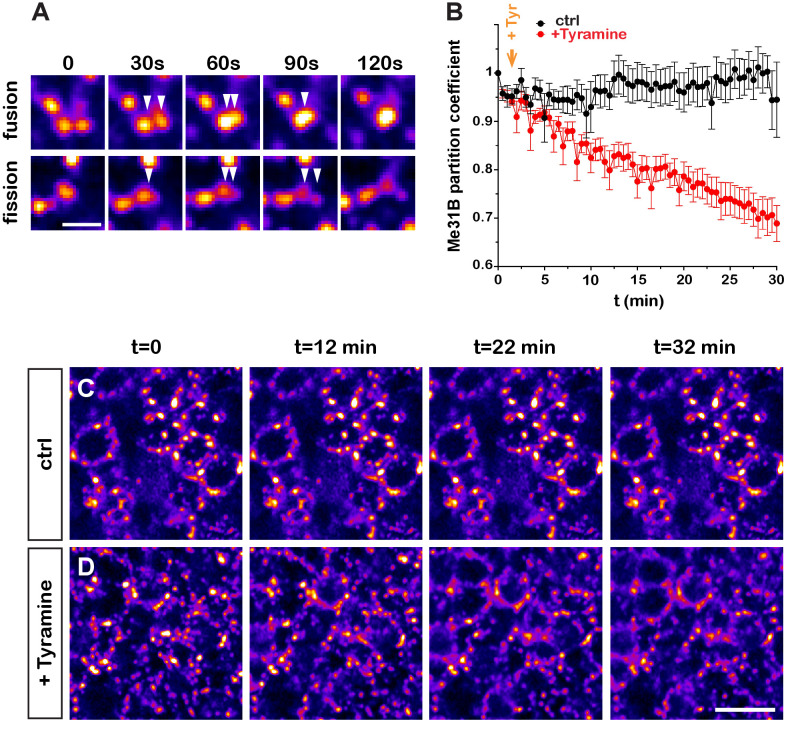


The article has been corrected accordingly.

