# Supplementary figures and images for: Tyramine induces dynamic RNP granule remodeling and translation activation in the Drosophila brain
Source: eLife. 2021 Apr 23;10:e65742. doi: 10.7554/eLife.65742 (PMC8064753; doi:10.7554/eLife.65742)

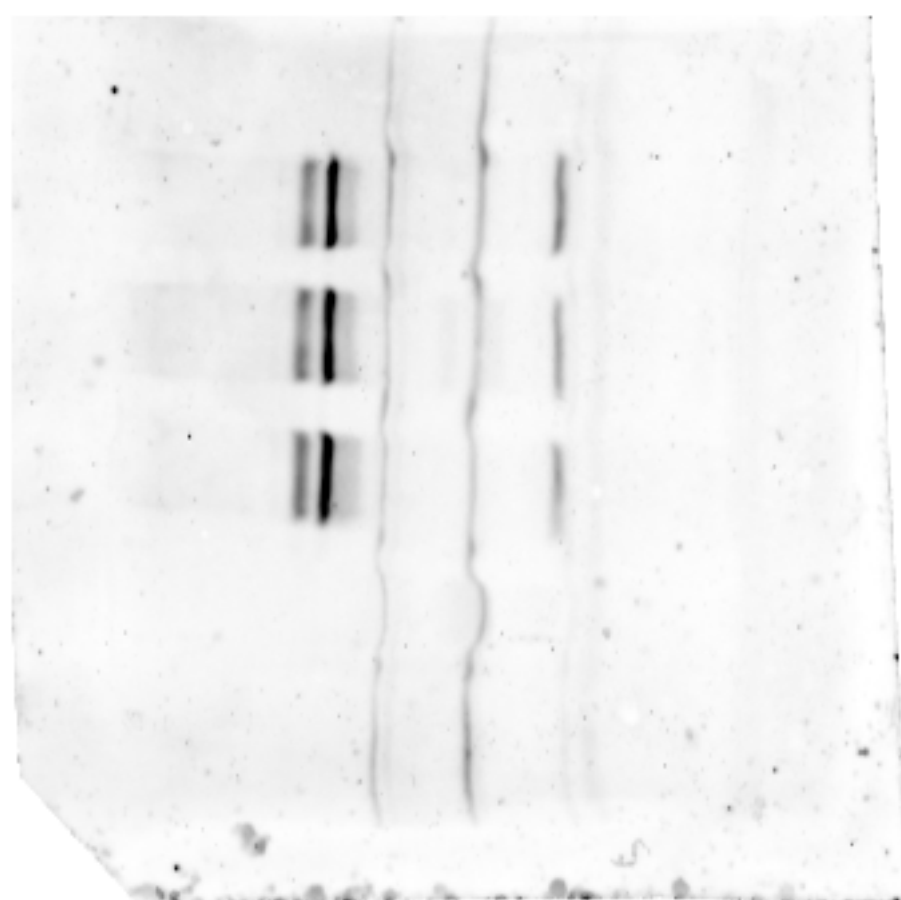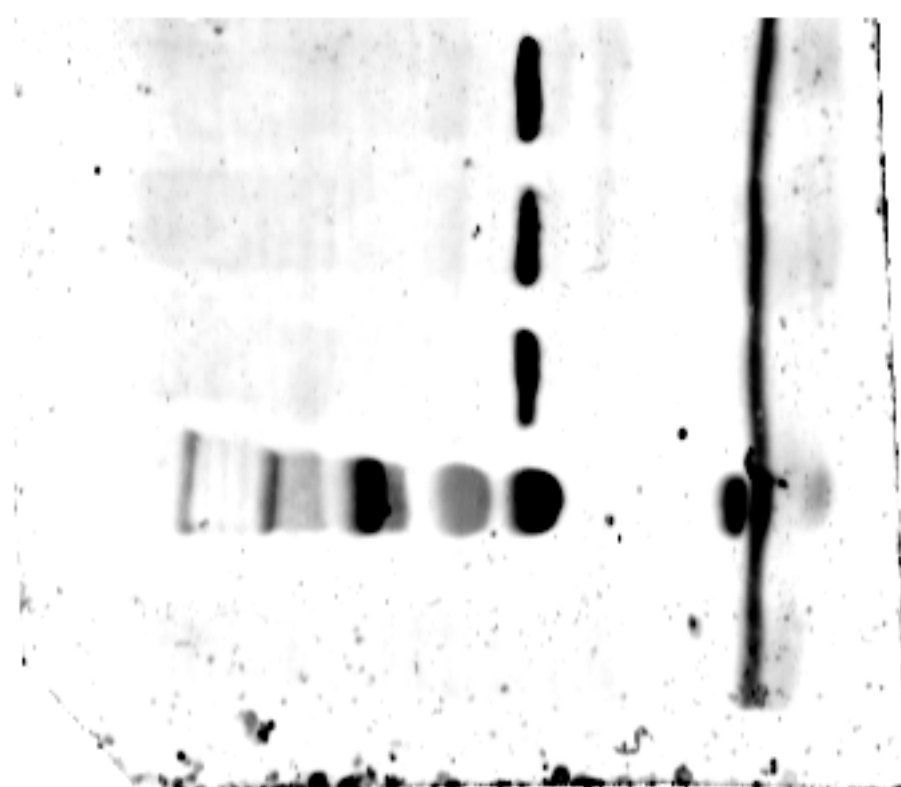

Supplement: Figure 1—figure supplement 2—source data 2. [file elife-65742-fig1-figsupp2-data2.pdf]

**bound**

**input**

**$\alpha$ -Flag**

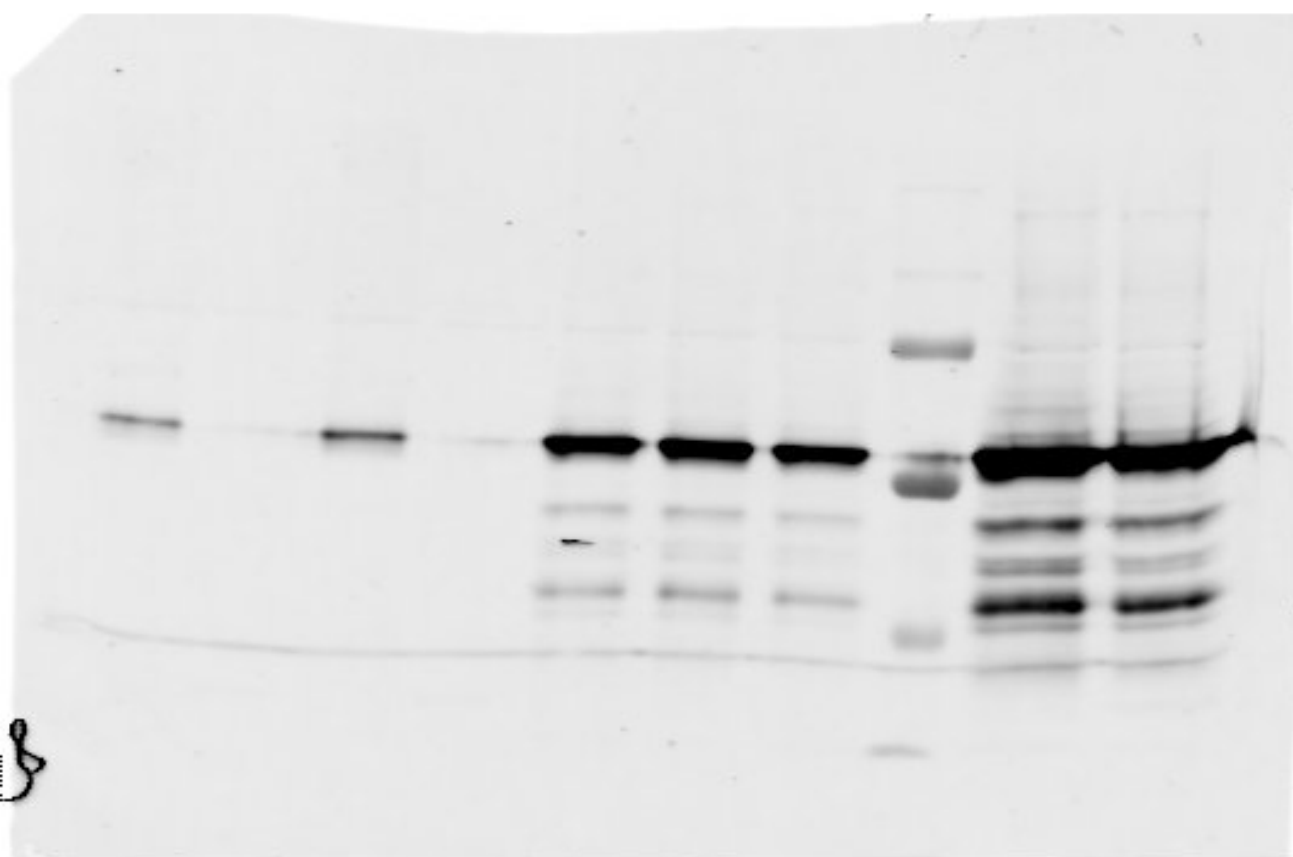

**$\alpha$ -GFP**

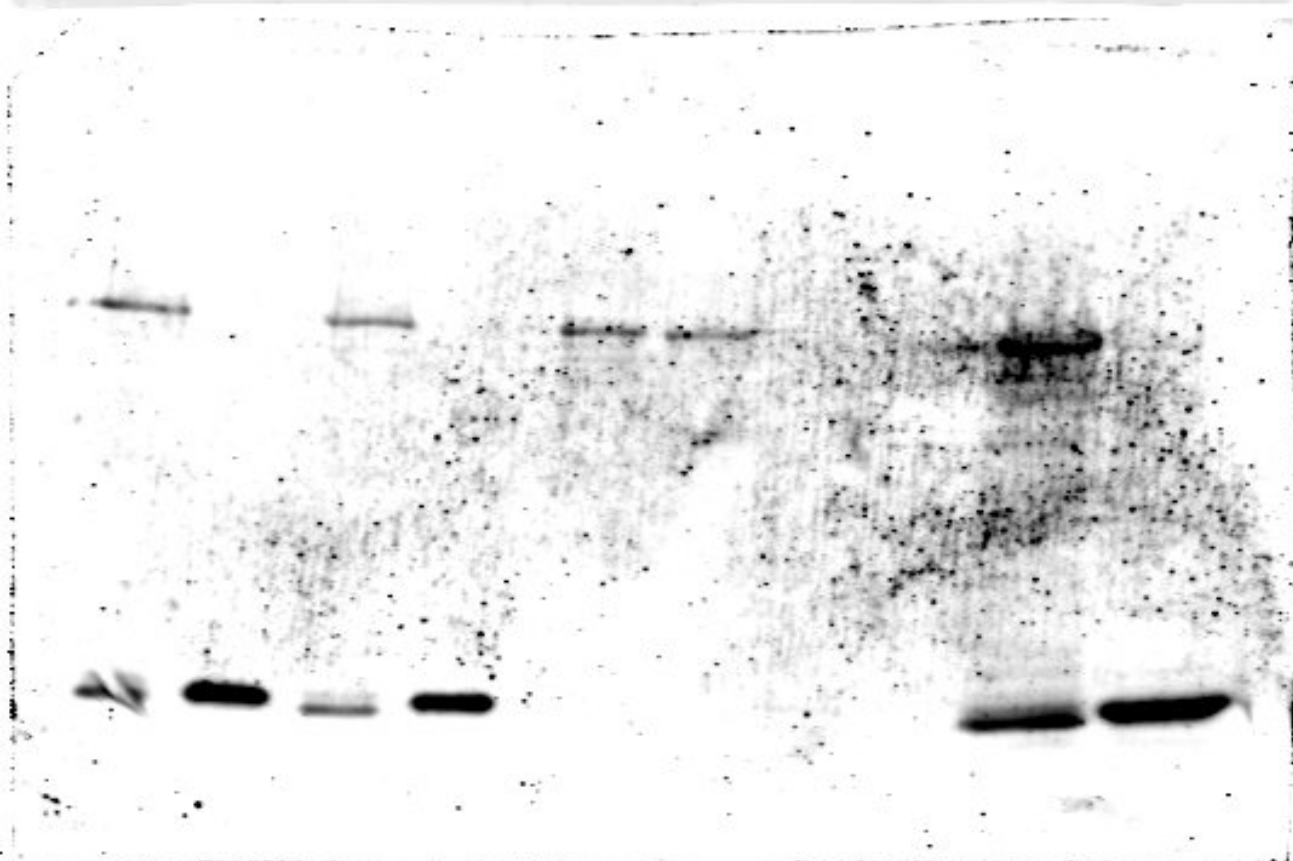

Supplement: Figure 4—source data 2. [file elife-65742-fig4-data2.pdf]
